# Dietary Patterns and Changes in Weight Status Among Chinese Men and Women During the COVID-19 Pandemic

**DOI:** 10.3389/fpubh.2021.709535

**Published:** 2021-12-13

**Authors:** Xiaoyue Xu, Alice F. Yan, Youfa Wang, Zumin Shi

**Affiliations:** ^1^Faculty of Medicine, School of Population Health, University of New South Wales, Kensington, NSW, Australia; ^2^Division of General Internal Medicine, Department of Medicine, Center for Advancing Population Science, Medical College of Wisconsin, Milwaukee, WI, United States; ^3^School of Public Health, Global Health Institute, Xi'an Jiaotong University Health Science Center, Xi'an, China; ^4^Human Nutrition Department, College of Health Sciences, QU Health, Qatar University, Doha, Qatar

**Keywords:** dietary pattern, weight status, COVID-19, Chinese, adults, survey

## Abstract

**Aims:** To identify dietary patterns during the coronavirus disease-2019 (COVID-19) pandemic and to examine their association with changes in weight status in the Chinese population.

**Methods:** The 2020 China COVID-19 cross-sectional survey is an anonymous 74-item survey administered *via* social media across 31 provinces in mainland China between April and May 2020. Dietary data were assessed by a Food Frequency Questionnaire and the changes in weight status were self-reported. Exploratory factor analysis using the principal component analysis method was applied to identify dietary patterns. The multinomial regression models were conducted, and forest plots were used to present the associations between dietary patterns and changes in weight status.

**Results:** Of a total of 10,545 adults (aged ≥18 years), more than half of participants reported to have weight gain, with 18.6% of men and 16.3% of women having weight gain >2.5 kg. Approximately 8% of participants reported to have weight loss, with 2.1% of men and 2.5% women having weight loss >2.5 kg. Two dietary patterns, namely, the modern and prudent dietary patterns, were identified during the COVID-19 pandemic. The modern dietary pattern was loaded heavily with soft drinks, fried foods, pickles, and inversely with fresh vegetables. The prudent dietary pattern was characterized by high intake of fresh fruits, vegetables, and inversely with soft drinks and fried food. The modern dietary pattern was positively associated with weight gain in men and women, while the prudent dietary pattern was negatively associated with both weight gain and loss in men and women during the COVID-19 pandemic.

**Conclusion:** Dietary patterns during COVID-19 are significantly associated with the changes in weight status, which may subsequently increase the risk of diet-related non-communicable disease among the Chinese population.

## Introduction

Coronavirus disease-2019 (COVID-19) is a pandemic induced by a severe acute respiratory syndrome coronavirus 2 (SARS-CoV-2) that has posed a public emergency of international concern (1, 2). As stated by the WHO on May 6, 2021, there have been over 154 million COVID-19 cases and 3 million deaths globally. The cumulative COVID-19 cases in China are 103,731 with 4,858 deaths (3).

Coronavirus disease-2019 is posing unprecedented challenges that have significant impact on human health, health system, health practitioners, economy, and society (2). To control the spread of COVID-19, China implemented vigorous measures of containment, mitigation, and suppression, such as travel restrictions, closing schools, and implementing remote work, particularly in the city of Wuhan (4). Although these measures have effectively controlled the spread of COVID-19, they have also substantially changed daily lives of peoples (5).

Several unhealthy lifestyles during the COVID-19 pandemic have been reported, such as less time spent on physical exercise and increased sedentary behavior (5). Stockpiling food due to grocery shopping restrictions reduced fresh food consumption (e.g., fruits and vegetables) and increased highly processed food consumption (e.g., snacks and junk foods) which are high in fats, sugars, and salt. In response to high levels of stress and anxiety, overeating, in particular consumption of unhealthy but “comfort foods” (e.g., food high in sugar) associated with higher energy intake, was common during the COVID-19 pandemic (5). In addition, data from China suggest a reduction in dietary diversity during the lockdown (6). These changes increased the risk for more severe complications of COVID-19 (7), also potentially increasing the risk of developing diet-related non-communicable diseases (NCDs), such as obesity and cardiovascular disease.

The association between weight gain and NCDs has been reported in previous literature (8, 9). For example, a 10-year follow-up study on the Nurses' Health Study and Health Professional Study shows that the incidence of common NCDs, such as diabetes, hypertension, heart disease, colon cancer, and stroke, increased based on the degree to which men and women were overweight (10). It is common knowledge that healthy diets can effectively prevent weight gain, obesity, and NCDs (9). Dietary patterns that illustrate the combined effects of dietary intake have been reported to link with diseases in the last two decades (11, 12). For example, the Mediterranean diet, which requires a high consumption of vegetables, fruits, legumes, cereals, fish, and a moderate intake of red wine, demonstrates a significant reduction in risk of mortality from cancer, development of cardiovascular diseases, presentation of depressive disorders, and incidence of Parkinson's disease and Alzheimer's disease (11), and it has been reported that people tended to have high adherence to Mediterranean diet during the COVID-19 pandemic in Spain (13). Our previous study on the Chinese population highlights the beneficial link between traditional dietary patterns (high intake of rice, pork, and vegetables) in preventing obesity, while modern dietary patterns (high intake of fast food and processed meat) related to the increased risk of obesity (14).

Although changes in lifestyle during the COVID-19 pandemic have been reported, dietary patterns during the pandemic and the link with changes in weight status have not been explored in detail yet. Therefore, the specific aims of this study were: (1) to identify dietary patterns among the Chinese adults during the COVID-19 pandemic, and (2) to examine the association between dietary patterns and weight change during the COVID-19 pandemic. Given that sex/gender differences in food choice and in energy and nutrient intake may affect weight status, we conducted the gender-specific analyses in this study. In addition, our previous study highlighted dietary consumption was affected by regions, which were mainly driven by economic and demographic factors (14). Therefore, we further tested whether there are interactions between dietary patterns and regional levels that may affect weight change status in the Chinese population.

## Methods

### Study Design and Participants

The 2020 China COVID-19 Survey is a cross-sectional, anonymous online survey that was administered *via* WeChat between April 25 and May 11, 2020. We selected this platform not only because the nation was under quarantine and we could only reach respondents online, but also because WeChat is leading social network of China, with more than 1 billion users, and the majority of Chinese adults use WeChat daily. To recruit a diverse sample of respondents, we started with convenience sampling and followed by a snowball sampling approach as an active means of recruitment (i.e., respondents themselves could recruit more respondents, such as their friends, family, co-workers, or acquaintances). The details of the study design and methods have been described in our previous publication (15). The survey method has been used in other surveys in China during the COVID-19 pandemic (6).

The survey questionnaire has 74 items with 150 study variables and eight themes: (1) awareness, knowledge, attitudes, and practices toward COVID-19; (2) personal experiences and impacts of COVID-19; (3) attitude toward government responses to COVID-19; (4) healthcare-seeking behaviors; (5) demographics characteristics; (6) lifestyle behaviors, (7) psychological well-being; and (8) health status with regard to NCDs and conditions. The details are outlined in our previous study (15). In total, there were 10,545 adults aged 18 years and over across 31 provinces in mainland China who participated in the survey. All procedures involving research study participants were approved by the Institutional Review Board of the Xi'an Jiaotong University, China, and participants provided consent.

### Measurements

#### Dietary Consumption

The items used for assessing dietary consumption and other health-related behaviors (e.g., physical activity) were drawn from the Kadoorie Study of Chronic Disease in China (KSCDC) (16) and the China Chronic Disease and Risk Factor (CCDRF) study, which were both developed by the China Center for Disease Control and Prevention (17). These surveys have been tested in the Chinese population and are considered as valid tools (15). The food frequency questionnaire (FFQ) consists of 16 food groups and beverages, such as (1) rice; (2) wheat flour foods; (3) fresh fruit; (4) fresh vegetables; (5) fruit and vegetable juice; (6) coarse grains; (7) fried foods; (8) soft drinks; (9) pickles; (10) fish and seafood; (11) poultry; (12) soymilk and soy products; (13) pork, beef, and lamb; (14) yogurt and other dairy products; (15) milk; and (16) eggs. Participants were asked to report how frequently they typically consumed each food during the lockdown using a 5-scale rating metric, i.e., every day, 4–6 days per week, 1–3 days per week, 1–3 per month, and never. In the analysis, we convert the frequency of consumption from 5-point scales to times per week.

#### Outcome: Changes in Weight Status

Changes in weight status were self-reported by asking the question of “how is your weight now compared to the pre-COVID period?” There are five scales in response to this question, i.e., unchanged, increased by 1–2.5 kg, increased by 2.5 kg or more, decreased by 1–2.5 kg, and decreased by 2.5 kg more.

#### Other Variables

Demographic variables were adapted from CCDRF (17). Education was allocated into three categories: low: primary school or below; medium: junior and/or high middle school; and high: bachelor's degree and higher. Residence was categorized into city, town, and rural. Alcohol drinking was allocated into three categories: current drinker, non-drinker, and ex-drinker. Smoker was also allocated into three categories: current smoker, non-smoker, and ex-smoker. Physical activity was measured with items adapted from the International Physical Activity Questionnaire (18). We further calculated weekly physical activity minutes, with >150 min physical activity being identified as adequate physical activity (15).

Participants were asked the questions of “Do you currently have an NCD or condition?” If so, participants were navigated to a question that prompted them to identify the specific disease or health condition. Status of currently having NCD or condition (Yes/No), such as heart disease, diabetes, and hypertension, was included as a covariable in the analysis.

### Statistical Analysis

The chi-squire tests were used to examine the statistical differences between characteristics and gender of participants. Exploratory factor analysis using the principal component analysis method was applied to identify dietary patterns (14). The frequency of a total of 16 food groups was included in the factor analysis.

Dietary patterns were identified based on the eigenvalue (>1), scree plot, factor interpretability, and the explained variance (>5%). Factors were rotated with varimax to improve the interpretability of factors and minimize the correlation between factors. Participants were assigned a pattern-specific factor score, which was calculated as the sum of the products of the factor loading coefficients and standardized daily intake of each food associated with that pattern. Factor loadings were included in the calculation of pattern scores. Factor scores were divided into four quartiles based on their distribution in each stratum. This method has been described in detail in our previous studies (14, 19).

Mean and SD of dietary pattern scores by status in weight change among men and women were described, with ANOVA used to present the statistical differences in means of dietary pattern scores across status in weight change by men and women. Mean and SD of food intake across quartiles of modern and prudent dietary patterns were presented, with ANOVA used to present the statistical differences for each dietary pattern by men and women.

We further grouped weight change status into three categories (i.e., unchanged, increased >1 kg, and decreased >1 kg), and then applied the multinomial regression models to examine the associations between dietary patterns and weight change status by gender. A forest plot with relative risk ratio (RRR) and 95% *CI* were used to present the results after adjustment for demographic and health behavior factors, as well as NCDs. Marginal plots were used to present the interaction term that impacted weight change status in men and women. Age, gender, education, residence, alcohol drinking, smoking, physical activity, hypertension, heart disease, and diabetes were adjusted as co-variables in the analysis.

All the analyses were performed using STATA 16.1 (Stata Corporation, College Station, TX, USA). Statistical significance was considered when *p* < 0.05.

## Results

The characteristics of participant are shown in Table 1. Of 10,545 participants, more than half of the participants were in the age range of 18–29 years, with a high education attainment level and self-reported as living in the city. There were 48.7% men and 83.3% women who were non-drinkers, 58.2% of men and 92.1% of women were non-smokers, and 64.2% of men and 55.1% of women had adequate physical activity. In terms of weight change, 39.8% of men and 42.1% of women had no weight change, 18.6% of men and 16.3% of women had weight increased by 2.5 kg or more, and 2.1% of men and 2.5% women had weight decreased by 2.5 kg more. The statistical differences were found across the characteristics and gender of participants (*p* < 0.05).

**Table 1:** Participants characteristics by men and women (*N* = 10,545).

**Characteristics**	**Men**	**Women**	***P*-value**
**Age group**		
18–29 years	2,382 (51.7)	2,607 (43.9)	<0.001
30–39 years	1,429 (31.0)	2,064 (34.8)	
40–49 years	512 (11.1)	840 (14.1)	
50–59 years	231 (5.0)	361 (6.1)	
60–80 years	51 (1.1)	68 (1.1)	
**Education**		
Low	100 (2.2)	91 (1.5)	0.008
Medium	1,686 (36.6)	2,299 (38.7)	
High	2,819 (61.2)	3,550 (59.8)	
**Residence**		
City	3,039 (66.0)	3,454 (58.2)	<0.001
Town	934 (20.3)	1,536 (25.9)	
Rural	632 (13.7)	950 (16.0)	
**Alcohol drinking**		
Current drinker	1,673 (36.3)	681 (11.5)	<0.001
Non–drinker	2,244 (48.7)	4,946 (83.3)	
Ex-drinker	688 (14.9)	313 (5.3)	
**Smoker**		
Current smoker	1,349 (29.3)	284 (4.8)	<0.001
Non-smoker	2,679 (58.2)	5,469 (92.1)	
Ex-smoker	577 (12.5)	187 (3.2)	
**Physical activity**		
Less than 150 min per week	1,647 (35.8)	2,666 (44.9)	<0.001
Greater than 150 min per week	2,958 (64.2)	3,274 (55.1)	
**Weight status**		
Unchanged	1,811 (39.8)	2,460 (42.1)	0.006
Increased by 1–2.5 kg	1,546 (34.0)	1,940 (33.2)	
Increased by 2.5 kg or more	846 (18.6)	954 (16.3)	
Decreased by 1–2.5 kg	249 (5.5)	345 (5.9)	
Decreased by 2.5 kg more	94 (2.1)	147 (2.5)	

**Figure 1:**
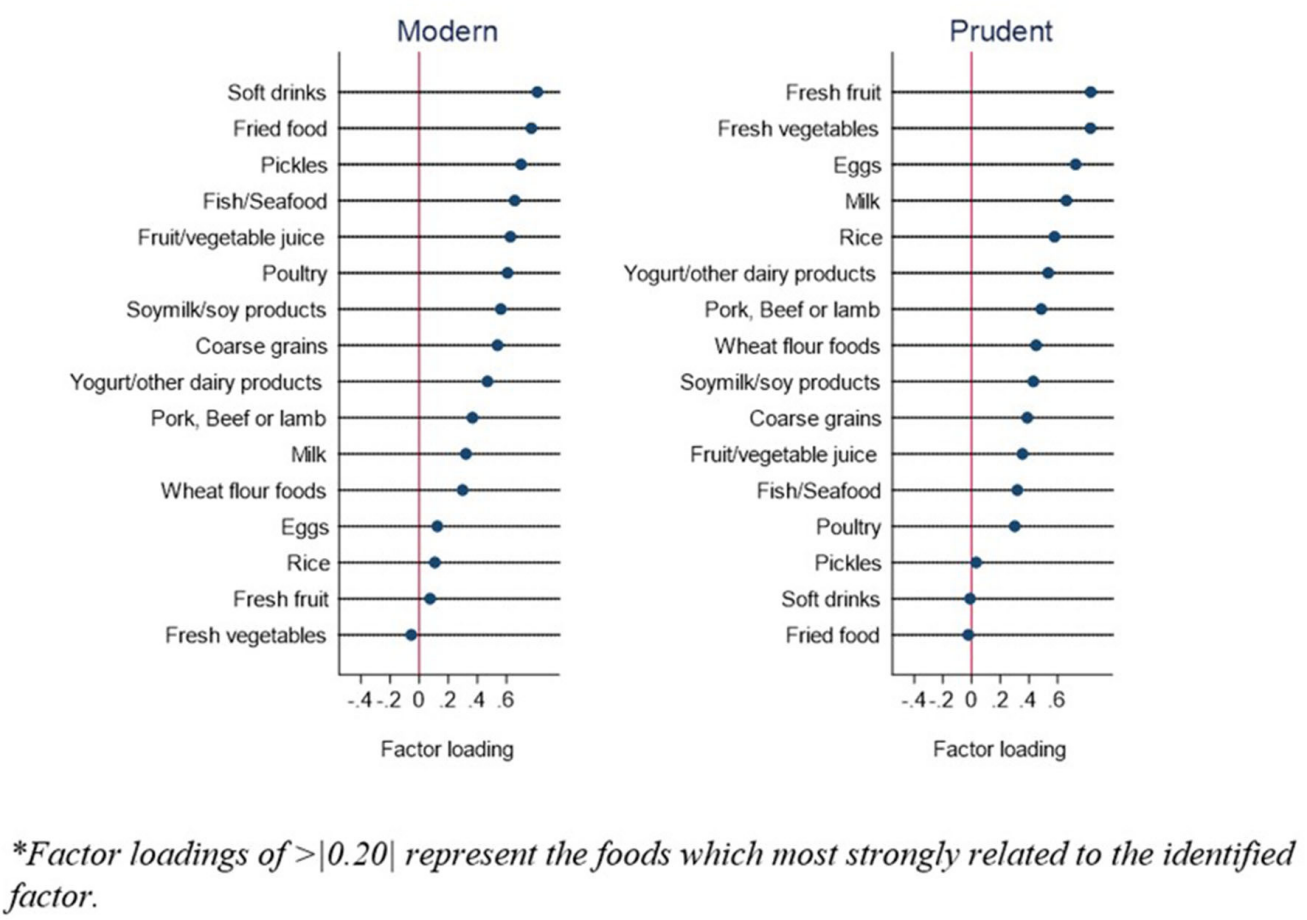
Factor loadings of two dietary patterns during COVID-19 pandemic*.

Two dietary patterns were obtained by using a factor analysis (Figure 1), namely, the modern and prudent dietary patterns. The modern dietary pattern (eigenvalue = 6.10) was loaded heavily with soft drinks, fried food, pickles, and inversely with fresh vegetables. The prudent dietary pattern (eigenvalue = 2.08) was characterized by high intake of fresh fruits and vegetables, and inversely on soft drinks and fried food. The two factors explained the 51.1% of variance in intake.

Mean of dietary pattern scores by weight status in men and women are shown in Table 2. Women had significant lower modern dietary pattern scores and higher prudent dietary pattern scores than men across weight status (*p* < 0.001), but no statistical differences were found between men and women among those who had their weight decrease by 2.5 kg or more (*p* = 0.08). The mean modern dietary pattern scores for men and women were 0.18 (SD, SD = 1.02) and −0.14 (SD = 0.96). The corresponding figures were −0.17 (SD = 1.03) and 0.13 (SD = 0.95) for prudent dietary pattern scores. Across city, town, and rural area, the mean modern dietary pattern scores were 0.02 (SD = 1.02), −0.03 (SD = 0.98), and −0.04 (SD = 0.97), and the mean prudent dietary scores were 0.08 (SD = 0.98), −0.003 (SD = 1.01), and −0.32 (SD = 0.99).

**Table 2:** Mean of dietary pattern scores by weight status in men and women.

	**Modern dietary pattern**		***P*-value**
	**scores, mean (SD)**		
	**Men**	**Women**	
**Weight status**					
Unchanged	0.14 (1.10)	−0.15 (0.99)	<0.001
Increased by 1–2.5 kg	0.23 (0.97)	−0.08 (0.96)	<0.001
Increased by 2.5 kg or more	0.24 (0.96)	−0.08 (0.97)	<0.001
Decreased by 1–2.5 kg	−0.04 (0.85)	−0.29 (0.85)	<0.001
Decreased by 2.5 kg or more	0.16 (1.11)	−0.41 (0.79)	<0.001
	**Prudent dietary pattern**		
	**scores, mean (SD)**		
	**Men**	**Women**	
**Weight status**			
Unchanged	−0.02 (0.99)	0.23 (0.93)	<0.001
Increased by 1–2.5 kg	−0.26 (1.06)	0.10 (0.96)	<0.001
Increased by 2.5 kg or more	−0.25 (1.05)	0.05 (0.95)	<0.001
Decreased by 1–2.5 kg	−0.36 (0.98)	−0.02 (0.97)	<0.001
Decreased by 2.5 kg or more	−0.30 (0.94)	−0.08 (0.93)	0.08

Food intakes across quartiles of modern and prudent dietary patterns are presented in Table 3. For the modern dietary pattern, there were significant lower consumption of fresh vegetables in quartile 4 (Q4) compared with Q1 (*p* < 0.01), with significant higher consumption for other food groups in Q4 compared with Q1, in particular, soft drinks and fried food among men and women (*p* < 0.001). For the prudent dietary pattern, there were significant lower consumption in soft drinks and fried food in Q4 compared with Q1 (*p* < 0.001), with significant higher consumption of other food groups in Q4 compared with Q1, in particular, fresh fruits and vegetables among men and women (*p* < 0.001).

**Table 3:** Food intakes across quartiles of modern and prudent dietary pattern by gender.

**Food (frequency)**	**Q1**		**Q2**		**Q3**		**Q4**		***P* for trend**
	**Mean**	**SD**	**Mean**	**SD**	**Mean**	**SD**	**Mean**	**SD**	
**(a)** Modern dietary pattern									
**Men**									
Soft drinks	0.24	0.51	0.67	0.97	1.71	1.64	4.60	2.31	<0.001
Fried food	0.36	0.56	0.77	0.84	1.57	1.52	4.38	2.47	<0.001
Wheat flour food	3.75	2.52	3.58	2.51	3.95	2.45	5.28	2.10	<0.001
Coarse grains	1.54	1.85	2.24	2.23	2.95	2.33	4.94	2.56	<0.001
Pork, beef or lamb	3.12	2.39	3.20	2.37	3.35	2.35	5.09	2.14	<0.001
Poultry	1.39	1.47	2.13	1.92	2.69	2.06	4.86	2.21	<0.001
Fish/Seafood	1.15	1.19	1.63	1.70	2.43	2.01	4.73	2.21	<0.001
Eggs	4.66	2.26	4.10	2.47	3.91	2.43	5.06	2.17	<0.001
Pickles	0.69	0.97	1.22	1.56	2.04	1.83	4.16	2.24	<0.001
Yogurt/dairy products	2.03	2.22	2.54	2.28	3.41	2.35	5.05	2.18	<0.001
Milk	3.13	2.68	3.22	2.62	3.70	2.57	5.10	2.21	<0.001
Fruit/vegetable juice	0.86	1.54	1.84	2.19	3.05	2.38	4.98	2.15	<0.001
Soymilk/soy products	1.44	1.69	2.31	2.15	3.14	2.27	4.96	2.17	<0.001
Rice	5.60	2.12	4.94	2.87	4.60	2.63	5.70	2.04	<0.001
Fresh fruit	5.00	2.25	4.11	2.61	4.05	2.59	5.16	2.17	<0.001
Fresh vegetable	6.23	1.44	4.91	2.43	4.49	2.49	5.31	2.11	<0.008
**Women**									
Soft drinks	0.18	0.38	0.51	0.79	1.32	1.54	4.51	2.38	<0.001
Fried food	0.37	0.56	0.86	1.12	1.57	1.67	4.17	2.54	<0.001
Wheat flour food	3.83	2.54	4.06	2.49	4.60	2.39	5.56	1.97	<0.001
Coarse grains	1.77	1.99	2.63	2.38	3.49	2.46	5.04	2.24	<0.001
Pork, beef or lamb	3.11	2.43	3.19	2.49	3.87	2.41	5.01	2.24	<0.001
Poultry	1.37	1.46	2.11	1.99	3.12	2.27	4.78	2.29	<0.001
Fish/Seafood	1.06	1.15	1.71	1.77	2.79	2.20	4.60	2.35	<0.001
Eggs	5.02	2.20	4.53	2.50	4.79	2.36	5.23	2.13	<0.001
Pickles	0.74	1.07	1.44	1.85	2.20	2.12	4.65	2.30	<0.001
Yogurt/dairy products	2.48	2.37	3.12	2.48	4.10	2.40	5.30	2.01	<0.001
Milk	3.38	2.74	3.74	2.75	4.46	2.58	5.35	2.07	<0.001
Fruit/vegetable juice	0.86	1.54	1.99	2.41	3.48	2.61	5.23	2.08	<0.001
Soymilk/soy products	1.77	1.91	2.58	2.32	3.59	2.37	5.19	2.10	<0.001
Rice	5.42	2.23	5.02	2.49	5.29	2.38	5.81	1.98	<0.001
Fresh fruit	5.59	2.00	4.88	2.52	5.02	2.39	5.47	2.03	0.002
Fresh vegetable	6.40	1.26	5.41	2.30	5.34	2.26	5.55	1.96	<0.001
**(b)** Prudent dietary pattern									
**Men**									
Soft drinks	1.81	1.82	2.08	2.33	3.10	2.99	1.35	2.04	<0.001
Fried food	1.82	1.88	1.96	2.26	3.00	2.90	1.28	1.76	<0.001
Rice	3.29	2.55	5.48	2.09	6.24	1.62	6.59	1.14	<0.001
Wheat flour food	2.79	2.20	4.16	2.30	5.14	2.31	5.52	2.09	<0.001
Coarse grains	1.91	1.87	2.75	2.40	4.09	2.69	4.44	2.45	<0.001
Pork, beef or lamb	2.24	1.92	3.75	2.24	4.88	2.35	5.08	2.18	<0.001
Poultry	1.95	1.73	2.86	2.18	4.01	2.61	3.61	2.53	<0.001
Fish/Seafood	1.73	1.68	2.43	2.15	3.72	2.61	3.46	2.47	<0.001
Eggs	2.22	1.75	4.41	1.99	5.76	1.77	6.42	1.11	<0.001
Pickles	1.98	1.85	2.41	2.29	3.43	2.83	1.80	2.18	<0.001
Yogurt/dairy products	1.78	1.65	3.07	2.25	4.61	2.50	5.25	2.20	<0.001
Milk	1.78	1.71	3.36	2.29	5.29	2.21	6.36	1.36	<0.001
Fruit/vegetable juice	1.77	1.79	2.48	2.40	4.03	2.80	4.23	2.73	<0.001
Soymilk/soy products	1.87	1.73	2.78	2.25	4.34	2.54	4.50	2.47	<0.001
Fresh fruit	1.90	1.58	4.45	1.87	6.34	1.22	6.85	0.62	<0.001
Fresh vegetable	2.57	1.92	5.52	1.51	6.67	0.82	6.95	0.30	<0.001
**Women**									
Soft drinks	1.38	1.75	1.45	2.05	1.97	2.70	0.91	1.73	<0.001
Fried food	1.63	1.94	1.65	2.04	2.07	2.58	0.96	1.51	<0.001
Rice	3.35	2.60	5.26	2.20	5.82	1.96	6.46	1.35	<0.001
Wheat flour food	2.99	2.28	3.99	2.34	4.77	2.40	5.46	2.19	<0.001
Coarse grains	1.81	1.91	2.50	2.29	3.37	2.65	4.11	2.56	<0.001
Pork, beef or lamb	1.85	1.82	3.23	2.28	4.31	2.46	4.81	2.34	<0.001
Poultry	1.69	1.75	2.27	2.05	3.22	2.52	3.18	2.49	<0.001
Fish/Seafood	1.38	1.61	1.82	1.91	2.87	2.49	3.01	2.41	<0.001
Eggs	2.26	1.87	4.26	2.07	5.64	1.79	6.54	1.10	<0.001
Pickles	1.89	2.00	2.27	2.32	2.56	2.66	1.56	2.06	<0.001
Yogurt/dairy products	1.75	1.75	2.80	2.20	4.04	2.52	5.16	2.26	<0.001
Milk	1.70	1.82	2.98	2.36	4.65	2.44	6.31	1.49	<0.001
Fruit/vegetable juice	1.48	1.78	1.95	2.32	3.00	2.88	3.74	2.92	<0.001
Soymilk/soy products	1.72	1.79	2.39	2.18	3.55	2.56	4.26	2.47	<0.001
Fresh fruit	1.99	1.63	4.78	1.88	6.35	1.17	6.92	0.41	<0.001
Fresh vegetable	2.71	1.99	5.71	1.43	6.69	0.81	6.95	0.37	<0.001

**Figure 2:**
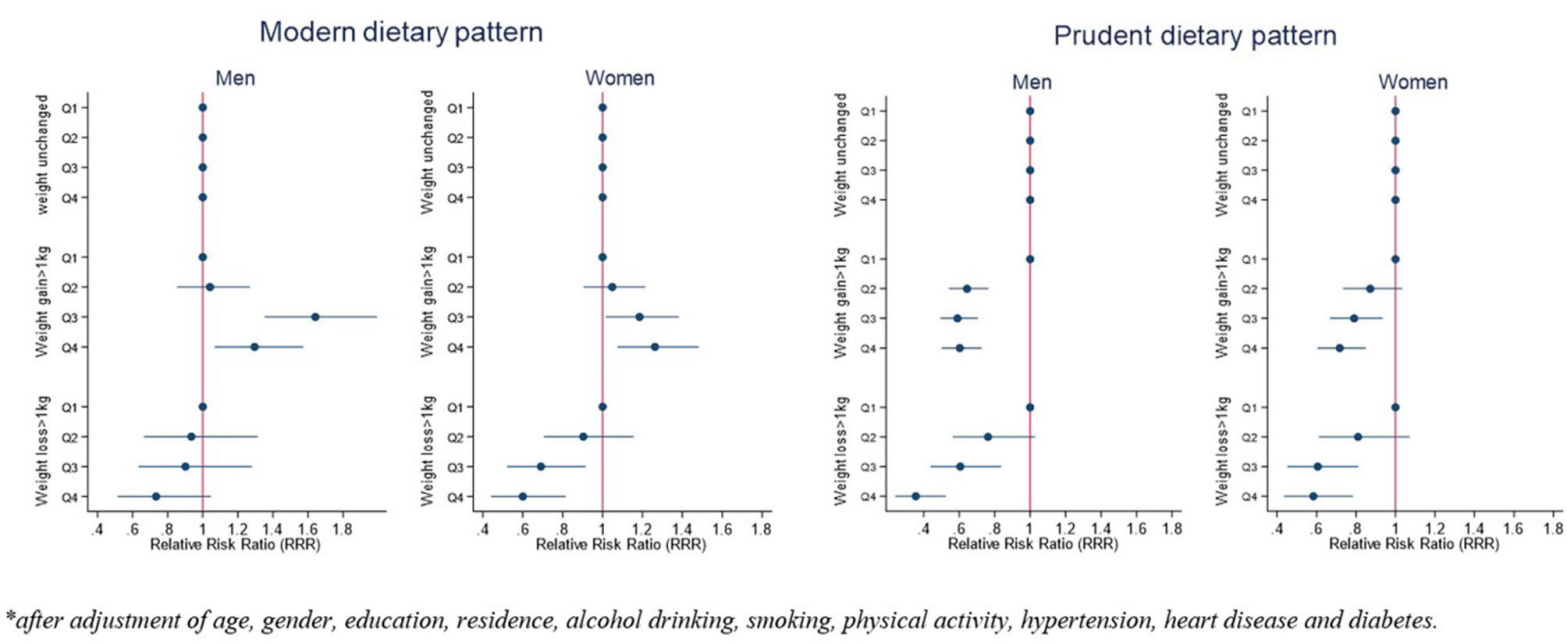
Associations between dietary patterns and weight change status by gender*.

The associations between two dietary patterns and weight status in men and women are shown in Figure 2. Compared with men with no weight change, the RRR (95% *CI*) of weight gain >1 kg across quartiles of modern dietary pattern among men was 1.00 (reference), 1.04 (0.86; 1.27), 1.64 (1.35; 1.99), and 1.30 (1.07; 1.57); the RRR (95% *CI*) of weight loss >1 kg across quartiles of the modern dietary pattern among men was 1.00 (reference), 0.94 (0.67; 1.31), 0.90 (0.64; 1.28), and 0.73 (0.52; 1.05). Compared with women with no weight change, the RRR (95% *CI*) of weight gain >1 kg across quartiles of the modern dietary pattern among women was 1.00 (reference), 1.05 (0.91; 1.21), 1.19 (1.02; 1.38), and 1.26 (1.08; 1.48); the RRR (95% *CI*) of weight loss >1 kg across quartiles of the modern dietary pattern among women was 1.00 (reference), 0.90 (0.71; 1.15), 0.69 (0.52; 0.91), and 0.60 (0.44; 0.81).

Compared with men with no weight change, the RRR (95% *CI*) of weight gain >1 kg across quartiles of the prudent dietary pattern among men was 1.00 (reference), 0.64 (0.54; 0.76), 0.59 (0.50; 0.70), and 0.60 (0.50; 0.70); the RRR (95% *CI*) of weight loss >1 kg across quartiles of the prudent dietary pattern among men was 1.00 (reference), 0.76 (0.57; 1.03), 0.60 (0.44; 0.83), and 0.36 (0.24; 0.52). Compared with women with no weight change, the RRR (95% *CI*) of weight gain >1 kg across quartiles of prudent dietary pattern among women was 1.00 (reference), 0.87 (0.74; 1.03), 0.79 (0.67; 0.93), and 0.71 (0.61; 0.85); the RRR (95% *CI*) of weight loss >1 kg across quartiles of the prudent dietary pattern among women was 1.00 (reference), 0.81 (0.61; 1.07), 0.61 (0.45; 0.81), and 0.58 (0.44; 0.78).

The interactions between dietary patterns and regional areas (city, town, rural) that impact weight change status were found in men and women. Men who lived in towns and had a higher modern dietary consumption were associated with a higher risk of weight gain. Women who lived in towns and rural areas and had a higher modern dietary consumption were associated with a higher risk of weight loss (Table 4). Similarly, men and women who had weight gain and lived in towns had a higher modern dietary pattern consumption (Supplementary Figure 1a), but a lower prudent dietary pattern consumption (Supplementary Figure 1b).

**Table 4:** Interaction between each dietary pattern and regional levels on weight change status, stratified by gender.

**Interaction**	RRR (95% CI)^*^			
**Dietary pattern and residence**	**Modern dietary pattern**		**Prudent dietary pattern**	
	**Men**	**Women**	**Men**	**Women**
**Weight unchanged**	1	1	1	1
**Weight gain** **>1 kg**				
Q1 # Urban	1	1	1	1
Q2 # Town	1.30 (0.80; 2.11)	1.06 (0.75; 1.49)	0.94 (0.61; 1.43)	0.83 (0.56; 1.23)
Q2 # Rural	0.90 (0.49; 1.65)	1.26 (0.83; 1.91)	0.77 (0.48; 1.23)	1.09 (0.71; 1.68)
Q3 # Town	**1.91 (1.18; 3.10)**	1.42 (1.00; 2.04)	1.36 (0.88; 2.10)	1.09 (0.73; 1.61)
Q3 # Rural	0.80 (0.45; 1.42)	1.03 (0.67; 1.58)	1.21 (0.72; 2.03)	1.43 (0.92; 2.21)
Q4 # Town	**1.67 (1.04; 2.67)**	1.45 (1.01; 2.09)	**1.65 (1.05; 2.59)**	0.85 (0.58; 1.25)
Q4 # Rural	0.84 (0.47; 1.50)	1.33 (0.85; 2.07)	1.63 (0.91; 2.92)	0.98 (0.61; 1.59)
**Weight loss** **>1 kg**				
Q1 # Urban	1	1	1	1
Q2 # Town	0.83 (0.33; 2.10)	1.40 (0.76; 2.60)	0.62 (0.25; 1.53)	1.21 (0.60; 2.45)
Q2 # Rural	0.92 (0.34; 2.50)	**2.53 (1.34; 4.77)**	1.01 (0.45; 2.27)	1.76 (0.91; 3.39)
Q3 # Town	1.34 (0.52; 3.42)	1.35 (0.67; 2.74)	1.63 (0.70; 3.81)	**2.34 (1.15; 4.75)**
Q3 # Rural	0.58 (0.21; 1.63)	1.60 (0.77; 3.30)	1.17 (0.43; 3.19)	2.00 (0.97; 4.11)
Q4 # Town	1.35 (0.53; 3.42)	**2.89 (1.43; 5.86)**	2.52 (0.95; 6.70)	1.14 (0.56; 2.34)
Q4 # Rural	0.95 (0.35; 2.60)	**2.79 (1.28; 6.11)**	2.86 (0.90; 9.11)	1.29 (0.57; 2.89)

* *Bold: p < 0.05*.

## Discussion

To the best of our knowledge, this is the first study to identify dietary patterns among the Chinese adults during the COVID-19 pandemic and to investigate their link with changes in weight status. Our results highlight how during the COVID-19 pandemic, more than half of participants reported to have weight gain, while ~8% of participants reported to have weight loss. Two dietary patterns, namely, the modern pattern and the prudent dietary pattern, were identified in the Chinese population during the COVID-19 pandemic. The modern dietary pattern is positively associated with weight gain in men and women, while negatively associated with weight loss in women. The prudent dietary pattern might prevent weight gain but might also affect weight loss.

Our results reveal that a significant proportion of Chinese adults reported to have weight gain during the COVID-19 pandemic, with ~20% of participants having weight gain >2.5 kg. This finding is consistent with the results from recent studies of other countries that report weight gain during the COVID-19 pandemic. For example, results from a cross-sectional study in Italy observed weight gain in 48.6% of a total of 3,533 participants (5). A study of 173 participants in United States showed 22% of participants had weight gain of 5–10 pounds (1 pound is equal to 0.45 kg), with reasons recorded, such as overeating, snacking after dinner, reduced physical activity, and inadequate sleep (20).

Weight gain may increase the risk of obesity and other serious diseases that include NCDs, such as coronary heart disease, stroke, type 2 diabetes, osteoarthritis, certain types of cancers, hypertension, and high cholesterol levels (21, 22). The concern has arisen that during the COVID-19 pandemic, the patients with NCDs delay accessing healthcare compared with patients with COVID-19. This is because the attention of health systems and resources are mainly focused on patients with COVID-19 and intensive care units (ICUs) might be overwhelmed by the serious pulmonary disease and its complications (4). Therefore, prevention of NCDs, such as maintaining a healthy weight during the COVID-19 pandemic, become extremely important.

In addition, our study found ~8% of participants reported to have weight loss, with more than 2% of them having weight loss >2.5 kg. Most existing studies on lifestyle and behavior changes emphasize the risks of weight gain and its consequence (5, 20), but few have reported on weight loss during the COVID-19 pandemic. We suspect that the reason for weight loss during the COVID-19 pandemic might be related to poor nutritional intake for people who are facing food supply issues during the indoor self-isolation restrictions. These factors have largely increased the risks of malnutrition on health, in particular, among older people.

The two dietary patterns, referred to in this study as the modern and prudent dietary patterns, that were identified during the COVID-19 pandemic in the Chinese population are similar to the dietary patterns identified during the pre-COVID-19 period (14, 23, 24). The key components of the modern dietary pattern are high consumption of soft drinks, fried food, pickles, and low consumption of fresh fruits and vegetables. The positive association between the modern dietary pattern and weight gain were consistent with current knowledge (14). The unhealthy food items, in particular, fried food and soft drinks, have been widely reported to be associated with weight gain and greater risk of obesity due to their high-calorie content favoring excess energy intake (25, 26).

The prudent dietary pattern among the Chinese population has similarities to the Mediterranean diet, which plays a protective role against weight gain (14). Interestingly, we found the prudent dietary pattern has a protective role against weight loss. The food items that are heavily loaded in the prudent dietary pattern include high consumption of fresh fruit, vegetable, egg, milk, rice dairy, pork, beef, lamb, fish and seafood, and low consumption of soft drinks and fried food. These food items are strongly aligned with the Chinese dietary guideline, which has been strongly encouraged as a balanced diet to prevent diseases for the Chinese population (27).

Our results show that compared with men, women had higher prudent dietary pattern scores. This implies that women are less likely to gain weight during the COVID-19 pandemic, tend to consume a healthy diet, and may subsequently have a lower risk than men of developing diet-related NCDs or conditions, such as diabetes, obesity, and hypertension. The evidence indicating the gender-specific dietary behavior during the COVID-19 pandemic is scarce. Findings regarding the interactions of dietary patterns and regional areas on weight change found in men and women were not surprising. Men and women who had weight gain and lived in towns had higher modern diet and lower prudent diet consumption patterns. The variety of dietary consumptions across regional areas was reported in our previous study (28). The present study aligns with our previous finding and highlights the interactions between dietary patterns and regional areas that impact weight change status in men and women during the COVID-19 lockdown. This may bring the disparity of future NCDs and their burden across regional areas in China. Therefore, public health nutrition intervention in prevention of NCDs during and after the COVID-19 lockdown should recognize these regional differences in dietary patterns.

To date, there is no scientific evidence that specific foods or supplements will prevent or cure COVID-19. A healthy balanced diet that supports overall well-being is a sound approach and has general health benefits aside from maintaining health during the COVID-19 pandemic (29). People are often encouraged to follow the general health eating advice to build a solid foundation for our immune system (30). Having a variety of foods can promote the intake of various immunity-friendly nutrients (such as vitamins, that include A, Bs, C, and D; and folate, iron, zinc, and selenium) and nourish our gut microbiota (using probiotics) (30). Our study results suggest that consuming a healthy balanced diet is associated with maintaining a healthy weight, which may potentially reduce the risk and burden of diet-related NCDs during the COVID-19 pandemic.

The strength of the present study includes a large and diverse population sample. The study is timely and provides strong evidence of a link between dietary patterns and changes in weight status. This evidence can be used to develop preventive measures against weight gain and loss during the COVID-19 pandemic. However, there are some limitations that must be noted. Principally is the sampling method. Convenience and snowball sampling methods are common in epidemiology studies when large numbers of participants are recruited, but they may have self-selection bias (15), which may affect the internal validity of the study. It might also be possible that our results may not represent the entire Chinese population. Second, our study is limited by using self-reported data, such as self-reported dietary consumption and weight status, which may have measurement bias that has overestimated or underestimated results. Third, our sample is relatively young with nearly half of the participants being between 18 and 29 years and only 17.7% being aged 40 years and over. This may have the implication that our results cannot be applied to the older Chinese people. Last, the number of food items included in the FFQ is relatively small, and the portion size of the food items was not measured.

In conclusion, our results show that during the COVID-19 pandemic, more than half of Chinese people in our sample reported to have weight gain, while a small proportion of people reported to have weight loss. Two dietary patterns were identified during the COVID-19 pandemic and its association with the changes in weight status were similar to the evidence found during the pre-COVID-19 period. We highlight the beneficial role of prudent dietary pattern in prevention of both weight gain and loss in Chinese men and women during the COVID-19 pandemic. In addition, we found women tended to consume a healthy and balanced diet, which may subsequently lead to a lower risk of developing diet-related NCDs than men. Our results indicate that a healthy diet prevent overweight and obesity during the COVID-19 pandemic. We encourage future studies to consider the gender differences in dietary consumption, as this may provide strong and novel evidence in prevention of diet-related NCDs.

## Data Availability Statement

The datasets presented in this article are not readily available because ethics restrictions. Requests to access the datasets should be directed to zumin@qu.edu.qa and youfawang@gmail.com.

## Ethics Statement

The studies involving human participants were reviewed and approved by the Institutional Review Committees of the Xi'an Jiaotong University, China. The patients/participants provided their written informed consent to participate in this study.

## Author Contributions

XX contributed to data analysis, data interpretation, drafted, and revised the manuscript. ZS, AY, and YW contributed to the study design, data collection, critically reviewed, and revised the manuscript. All authors read and approved the final manuscript.

## Funding

This project was supported in part by research grants from the China Medical Board (Grant number: 16-262), the National Key Research and Development Program of China (Grant Number: 2017YFC0907200 and 2017YFC0907201), the University Alliance of the Silk Road (Grant number: 2020LMZX002), and the Xi'an Jiaotong University Global Health Institute. XX was supported by the Australia National Heart Foundation post-doctoral fellowship (Award number: 102597), and University of New South Wales Scientia Program.

## Conflict of Interest

The authors declare that the research was conducted in the absence of any commercial or financial relationships that could be construed as a potential conflict of interest.

## Publisher's Note

All claims expressed in this article are solely those of the authors and do not necessarily represent those of their affiliated organizations, or those of the publisher, the editors and the reviewers. Any product that may be evaluated in this article, or claim that may be made by its manufacturer, is not guaranteed or endorsed by the publisher.
