# Supplementary material for: Dietary Patterns and Changes in Weight Status Among Chinese Men and Women During the COVID-19 Pandemic
Source: Front Public Health. 2021 Dec 13;9:709535. doi: 10.3389/fpubh.2021.709535 (PMC8710477; doi:10.3389/fpubh.2021.709535)
Supplement: Supplementary file 1 [file Data_Sheet_1.docx]

Supplementary Material

**Supplementary Figure 1. Average marginal effects of each dietary pattern with 95% CIs on weight status change**

1. **Modern dietary pattern b) Prudent dietary pattern**

**
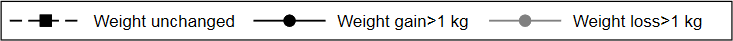
**
